# Complete mitochondrial genome sequence of Tosa-Jidori sheds light on the origin and evolution of Japanese native chickens

**DOI:** 10.5713/ajas.19.0932

**Published:** 2020-04-13

**Authors:** Sayed A.-M. Osman, Masahide Nishibori, Takahiro Yonezawa

**Affiliations:** 1 Laboratory of Animal Genetics, Department of Animal Life Science, Graduate School of Integrated Sciences for Life, Hiroshima University, Hiroshima 739-8528, Japan; 2 Department of Genetics, Faculty of Agriculture, Minia University, El Minia, Eg-61517, Egypt; 3 Faculty of Agriculture, Tokyo University of Agriculture, Atsugi, Kanagawa 243-0034, Japan

**Keywords:** D-loop, Jidori, Mitogenome, Phylogeny, Chicken, *Gallus gallus*

## Abstract

**Objective:**

In Japan, approximately 50 breeds of indigenous domestic chicken, called Japanese native chickens (JNCs), have been developed. JNCs gradually became established based on three major original groups, “Jidori”, “Shoukoku”, and “Shamo”. Tosa-Jidori is a breed of Jidori, and archival records as well as its morphologically primitive characters suggest an ancient origin. Although Jidori is thought to have been introduced from East Asia, a previous study based on mitochondrial D-loop sequences demonstrated that Tosa-Jidori belongs to haplogroup D, which is abundant in Southeast Asia but rare in other regions, and a Southeast Asian origin for Tosa-Jidori was therefore suggested. The relatively small size of the D-loop region offers limited resolution in comparison with mitogenome phylogeny. This study was conducted to determine the phylogenetic position of the Tosa-Jidori breed based on complete mitochondrial D-loop and mitogenome sequences, and to clarify its evolutionary relationships, possible maternal origin and routes of introduction into Japan.

**Methods:**

Maximum likelihood and parsimony trees were based on 133 chickens and consisted of 86 mitogenome sequences as well as 47 D-loop sequences.

**Results:**

This is the first report of the complete mitogenome not only for the Tosa-Jidori breed, but also for a member of one of the three major original groups of JNCs. Our phylogenetic analysis based on D-loop and mitogenome sequences suggests that Tosa-Jidori individuals characterized in this study belong to the haplogroup D as well as the sub-haplogroup E1.

**Conclusion:**

The sub-haplogroup E1 is relatively common in East Asia, and so although the Southeast Asian origin hypothesis cannot be rejected, East Asia is another possible origin of Tosa-Jidori. This study highlights the complicated origin and breeding history of Tosa-Jidori and other JNC breeds.

## INTRODUCTION

Given the unique geographical location of Japan in the East Asian peripheral region, with water surrounding it on all sides, Japanese native chicken (JNC) breeds of *Gallus gallus* have been profoundly influenced by continental breeds, but at the same time, they have developed indigenously. There are approximately 50 breeds of JNCs [1]. Until 1867, most of them were developed for their special plumage, crowing and cockfighting, while subsequently, from the end of the 19th century to the early stages of the 20th century, some new breeds were created to produce eggs and/or meat.

Most of today’s JNC breeds were established from members of three major original groups or breeds: “Jidori”, “Shoukoku”, and “Shamo” [2]. Jidori does not specify a breed, but is a generic name used for various local primitive chickens whose ancestors are thought to have been introduced to Japan in the Yayoi period (300 BC through 300 AD) via Korea. The Jidori group includes the three well-known breeds Tosa-Jidori, Gifu-Jidori, and Mie-Jidori. Shoukoku is a breed originating in China that has developed as an aesthetic breed, which was introduced to Japan in the Heian period (794 through 1192 AD). Shoukoku and its derivative breeds such as Onaga-dori and Toutenkou constitute the Shoukoku group. Shamo is thought to have been derived from a Malay-type chicken from Thailand during the early 17th century (Edo period). The Shamo group comprises seven breeds, namely Oh-Shamo, Ko-Shamo, Yamato-Shamo (Yamato Gunkei), Yakido, Kinpa, Nankin-Shamo, and Echigo-Nankin-Shamo. However, the details of how these groups reached Japan are obscure [3–6].

Population genetic analysis of JNC breeds has been largely carried out by Oka et al [6]. However, their study was based on mitochondrial DNA D-loop sequence only, and they used limited numbers of global chicken samples other than JNC. Most studies of chicken mitochondrial DNA have also relied on D-loop sequencing [7–12]. The small size (about 1,230 bp) of the D-loop limits the resolution of the maternal phylogeny. Moreover, the mutation rate in this region is higher than that in the coding region, which can blur the structure of the matrilineal genealogy [13]. Recently, fine-grained analyses have used the mitogenome to reconstruct the history of chicken domestication [13,14]. These updated phylogenies provide new insights into the origins and history of domestication. Miao et al [13] noted some discordances between D-loop and complete mitogenome sequence data. Several sequences which previously fell into haplogroup D, based on D-loop data, were assigned to haplogroups Y and C based on mitogenome data. Moreover, a sequence that was previously classified as a basal branch in haplogroup C was redefined as belonging to the new haplogroup X based on mitogenome data. Also, Huang et al [14] found that some lineages of red junglefowl from Thailand, previously assigned to haplogroup C* based on D-loop sequences, were re-clustered as a new haplogroup V based on their mitogenomes. These lineages of red junglefowl were situated at the basal branch of haplogroups C and D, which were defined according to Miao et al [13]. To achieve the highest possible level of molecular resolution, complete mitogenome sequences are necessary [13].

Tosa-Jidori is a small type of Jidori that was already in Kochi Prefecture by the end of the Edo period (1603 through 1867) [2]. Tosa-Jidori together with 16 other JNC breeds are designated as “natural national monuments” by the Japanese Government [3]. Oka et al [6] reported previously that Tosa-Jidori belonged to haplogroup D, which is abundant in Southeast Asia but rare in other regions. Therefore, they concluded that Tosa-Jidori is of Southeast Asian origin, although, as noted above, their study was based on the mitochondrial D-loop region only.

Although Tosa-Jidori is one of the oldest and most impor tant breeds of JNCs, its origin is still unclear. Understanding the source of Tosa-Jidori will thus shed more light on the origin and routes of introduction of JNC breeds. The aim of this study was therefore to determine the phylogenetic position of Tosa-Jidori, based on complete mitochondrial D-loop and mitogenome sequences, and to clarify its phylogenetic relationships, possible maternal origin and routes of introduction into Japan.

## MATERIALS AND METHODS

### Sample collection and DNA isolation

Because the taxonomic characters within *Gallus* are based on male morphological features, DNA was extracted from peripheral blood of one male from each of six different strains (910, 921, 911, 922, 51, and Tsuchimoto) of the Tosa-Jidori breed kept at Kochi Prefectural Livestock Experiment Station (KPLES), using the phenol/chloroform method as described by Green and Sambrook [15]. These blood samples were collected by author (M.N.) and staff of the KPLES under the ethics guidelines of the KPLES and Hiroshima University.The sequenced birds are parent stocks with no kin relationships among individuals. Blood samples were collected from the brachial vein, using heparinized syringes. The samples were transferred to ice and then kept frozen at −40°C for later analysis.

### Amplification and sequencing

We determined the complete sequence of the mitochondrial D-loop region for one individual from each strain (910, 921, 911, 922, 51, and Tsuchimoto), following the procedure described by Osman et al [10], and the individual from strain 921 was chosen for determination of the complete mitochondrial genome sequence.

Subsequently, the complete mitogenome sequence was determined by the following procedures. Mitochondrial DNA fragments were specifically amplified using a KOD-FX kit (KOD-FX polymerase, Toyobo, Osaka, Japan) with purified DNA as a template and LA-PCR primer sets, following the procedure described in previous studies [16,17]. Two primer sets were used for LA-PCR: the first primer set for 16S ribosomal RNA (16S rRNA) was as follows: LA16SF, 5′-CCTACGTGATCTGAGTTCAGACCGGAGCAATC CAG-3′; and LA16SR, 5′-TGCACCATTAGGTTGTCCT GATCCAACATCGAGGT-3′. This set was designed to amplify a fragment of about 16 kilobase pairs (kb) from downstream of 16S rRNA to upstream of 16S rRNA, spanning the cytochrome b (*Cytb*) gene. The second primer set for the Cytb gene was as follows: LACytbF, 5′-TACACGAATCAGGCT CAAACAACCCCCTAGGCATC-3′; and LACytbR: 5′-AG ATACAGATGAAGAAGAATGAGGCGCCGTTTGCG-3′. This primer set was also designed to amplify a fragment of about 16 kb from downstream of *Cytb* to upstream of *Cytb*, spanning 16S rRNA. Polymerase chain reaction (PCR) amplifications were carried out in 20 μL reaction volumes containing 25 ng genomic DNA, 1× PCR buffer, 0.4 mM each dNTP, 0.3 μM each primer and 0.4 U of Taq DNA polymerase. The LA-PCR comprised an initial denaturation at 94°C for 2 min, followed by 30 cycles consisting of 10 s denaturation at 98°C, and 17 min annealing and extension at 68°C, using a GeneAmp PCR System 9700 (Life Technologies, Foster City, CA, USA). Amplified fragments were isolated by agarose gel electrophoresis as described by Osman et al [18], and then used for segmental amplification of the mitogenome, with 37 primer sets as described by Nishibori et al [17] as well as the following three new primers for Tosa-Jidori: Tosa 487R 5′-AAGGCAAGTAGGGCGAGGGGTGTA-3′, Tosa 1048F 5′-CTACCGCATAAAATCCCTCAAACTA-3′ and Tosa 1886F 5′-AGCCTGTTCTATAATCGATAAT-3′. PCR amplifications were carried out in 20 μL reaction volumes containing 25 ng of 16 kb fragment as templates, 1× PCR buffer, 0.2 mM each dNTP, 2.5 mM MgCl_2_, 0.2 μM each primer and 0.4 U of Taq DNA polymerase (BIOTaq HS DNA polymerase, BIOLINE, Boston, MA, USA). Reaction conditions were: 95°C (9 min), 35 cycles of 95°C (30 s), 58°C (30 s), and 72°C (1 min), and a final extension step at 72°C (5 min), using a GeneAmp PCR System 9700. The PCR products were electrophoresed on a 1% agarose gel (Nippon Gene, Osaka, Japan), and visualized using an ultraviolet transilluminator after staining with ethidium bromide. The mitogenome fragments obtained through segmental amplification were sequenced using ExoSAP-IT (Amersham Biosciences, Buckinghamshire, UK) and an ABI Prism BigDye Terminator v 3.1 Cycle Sequencing Kit (Life Technologies, USA) and the corresponding primer. Labeled mitogenome fragments were purified using a BigDye XTerminator Purification Kit (Life Technologies, USA) and analyzed on a Model 3130x1 genetic analyzer (Life Technologies, USA). To obtain reliable sequence data, each mitogenome strand was sequenced at least twice. Sequences thus obtained were assembled into the full-length mitogenome using GPROF software (ver. 2.0; Software Development, Tokyo, Japan). The full-length mitogenome sequence was compared with that of another chicken breed, White Leghorn (DDBJ/EMBL/GenBank accession no. AP00 3317) reported by Nishibori et al [19] using the GENETYX program package (ver.11; Software Development).

### Phylogenetic analysis

Phylogenetic trees were reconstructed by the maximum parsimony (MP) and maximum likelihood (ML) methods, based on 133 chickens consisting of 86 mitogenome sequences (Tosa-Jidori, determined in this study, and 85 mitogenomes collected from NCBI) as well as 47 D-loop sequences (five Tosa-Jidori individuals determined in this study and 42 haplotypes described by Oka et al [6]: A01–A10, B01–B08, C01–C08, D01–D09, E01–E04, F01–F02, and G01) (Supplementary Table S1).

To take account of the different tempo and mode of mo lecular evolution between the mitogenome and D-loop as well as the effect of missing sites, two types of alignment were used for reconstructing phylogenetic trees in this study. The first type of alignment consists of the nucleotide sequences of the D-loop only (1,233 bp), and the second consists of the nucleotide sequences of the whole mitogenome. Regarding the 47 D-loop sequences (five from this study and 42 from Oka et al [6]), non-D-loop regions were treated as missing data. For the second type of alignment, whole sites of 16,797 bp were involved in reconstructing the phylogenetic trees based on 86 mitogenome and 47 D-loop only sequences. However, KR347464 (Jinhu Wufeng Chicken) has a considerable number of private mutations between 11,839th and 11,964th site, and this region (126 bp in total length) was therefore excluded from KR347464 in the analysis (in contrast to the 21 singleton mutation site found in KR347464, no mutations were found in the other 85 mitogenomes in this region).

The ML trees were inferred by RAxML ver. 8.2.10 [20] with the GTR+I+Γ model. The MP trees were inferred by MEGA6 [21] with the tree bisection regrafting algorthism. The identical sequences were excluded from the MP tree inference. Bootstrap values were estimated with 1,000 replications. Nomenclatures of the haplogroups followed those of Miao et al [13].

## RESULTS AND DISCUSSION

Genetic diversity and relationships among chicken breeds have been studied widely in the past decade using mitochondrial sequences [6–12,18,22]. This current study is the first to address the origin of the Tosa-Jidori breed, indigenous to Kochi Prefecture, using complete D-loop and mitogenome sequences. Complete D-loop sequences of six blood samples (Tosa 1–Tosa 6) from six different strains of the Tosa-Jidori breed (accession numbers LC507812–LC507817) were determined, and three haplotypes were identified in the present study (Supplementary Table S2). We found six sites with variant nucleotides, which were all transitions.

**Figure 1:**
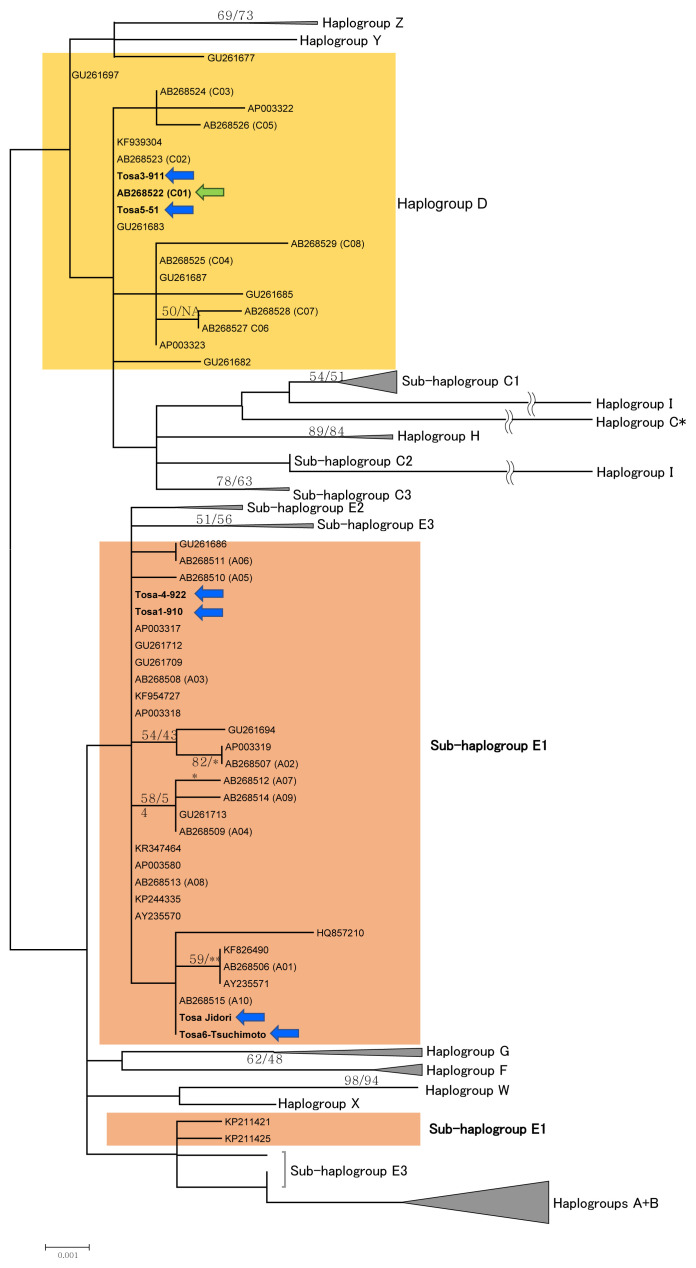
A maximum likelihood (ML) phylogenetic tree of chickens (the log-likelihood score is −2,846.2) based on complete mitochondrial D-loop sequences of 133 individuals. The gaps were treated as missing nucleotides, and all sites (1,233 bp) were included in this analysis. The detailed phylogenetic relationships were shown only for the haplogroup D (indicated in yellow color) and the sub-haplogroup E1 (indicated in brown color), and other haplogroups (or sub-haplogroups) were compressed. The detailed relationships among all sequences are shown in Supplementary Figure S1. Nodal numbers indicate the bootstrap values with 1,000 replications (ML-bootstrap/MP-bootstrap). If both of ML and MP bootstrap values are lower than 50%, they were not indicated. NA means the monophyletic relationships were not obtained in the MP analysis. ** Means monoplyletic relationships due to identical sequences. The names of operational taxonomic units (OTUs) are indicated by NCBI accession numbers. Haplotype names defined by Oka et al [6] (see Supplementary Table S1) are also indicated in parentheses after the relevant accession numbers. Nomenclature of the haplogroups follows Miao et al [13]. Six sequences of Tosa-Jidori (in this study) as well as Tosa-Jidori studied by Oka et al [6] (accession number AB268523-C02) are indicated by green and blue arrows, respectively. The branch lengths are proportional to the nucleotide substitution rate. However, the branches of haplogroups C* and I are partially omitted due to their long branch lengths.

First, we constructed an ML tree (Figure 1, Supplementa ry Figure S1) using the complete D-loop region sequences of our samples of Tosa-Jidori (Tosa 1–Tosa 6) with that of Oka et al [6], and using the reference sequence of Miao et al [13], which represent the different regions of chicken domestication. We found that two individuals (Tosa 3 and Tosa 5) of our samples shared the same haplogroup with Oka et al [6] (Haplogroup D), while the other four (Tosa 1, Tosa 2, Tosa 4, and Tosa 6) individuals were located in sub-haplogroup E1 (Figure 1, Supplementary Figure S1). The MP tree showed essentially identical branching patterns to those of the ML tree (data not shown). There are partial contradictions regarding the origin of Tosa-jidori breed based on D-loop region sequences between our study and that of Oka et al [6], so we determined the mitogenome sequence of Tosa-jidori to confirm the position of Tosa-jidori.

As noted in the introduction, fine-grained analyses have used the complete mitogenome to reconstruct the history of the domestication of animals such as chickens [13,14]. These updated phylogenies provide new insights into the origins and history of domestication. With the aim of addressing these questions for Tosa-Jidori, we selected one sample (Tosa 2; 921 strain) for determination of the complete mitogenome sequence as described by other researchers using different bird species (Japanese quail [16]; chickens [17,19,23]; rock ptarmigans [24]).

**Figure 2:**
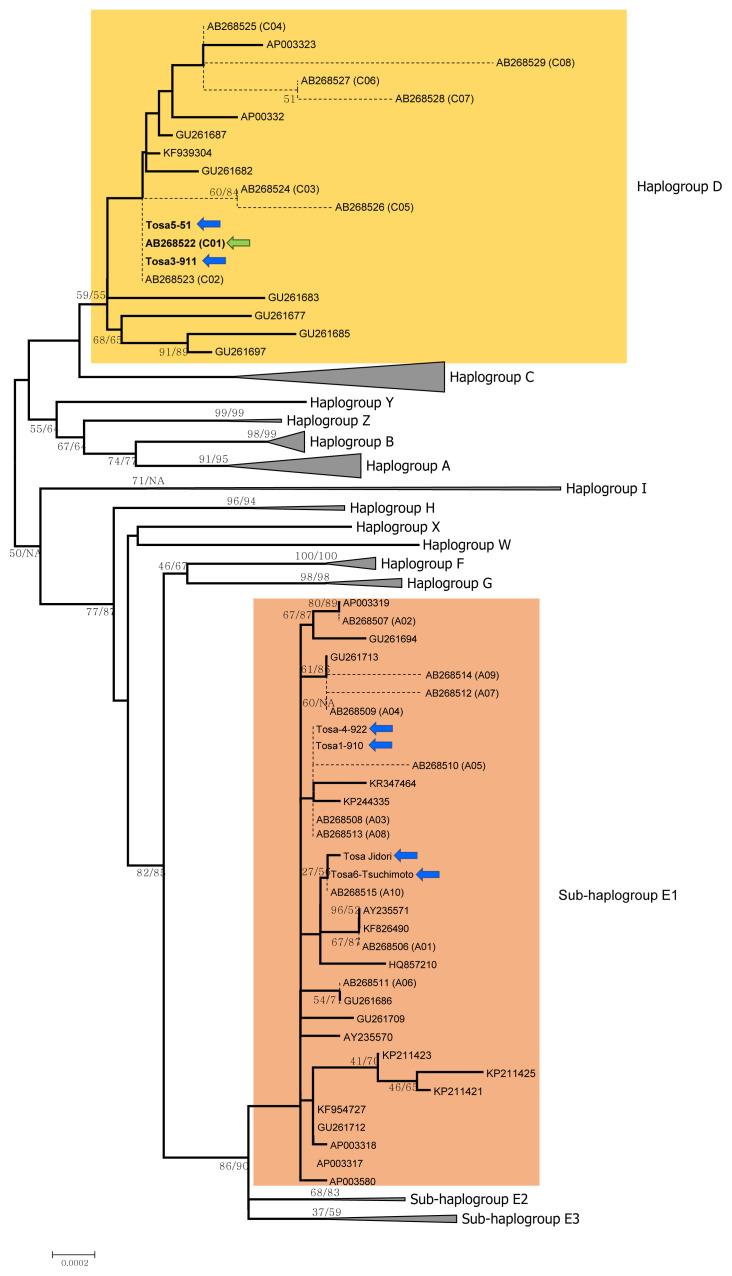
A maximum likelihood (ML) phylogenetic tree of chickens (the log-likelihood score is −27,799.9). The gaps were dealt as the missing nucleotide, and all sites (16,797 bp) were involved in this analysis. The detailed phylogenetic relationships were shown only for the haplogroup D (indicated in yellow color) and the sub-haplogroup E1 (indicated in brown color), and other haplogroups (or sub-haplogroups) were compressed. The detailed relationships among all sequences are shown in Supplementary Figure S2. Nodal numbers indicate the bootstrap values with 1,000 replications (ML-bootstrap/MP-bootstrap). If both of ML and MP bootstrap values are lower than 50%, they were not indicated. NA means the monophyletic relationships were not obtained in the MP analysis. Complete mitogenome sequences are indicated by solid lines and the shorter complete mitochondrial D-loop sequences are indicated by thin dashed lines. The names of operational taxonomic units (OTUs) are indicated by NCBI accession numbers. Haplotype names defined by Oka et al [6] (see Supplementary Table S1) are also indicated in parentheses after the relevant accession numbers. Nomenclature of the haplogroups follows Miao et al [13]. Six sequences of Tosa-Jidori (in this study) as well as Tosa-Jidori studied by Oka et al [6] (accession number AB268523-C02) are indicated by green and blue arrows, respectively. The branch lengths are proportional to the nucleotide substitution rate.

The nucleotide sequence of the whole mitogenome of Tosa- jidori was determined (accession number LC082227), and its length is 16,787 bp. To verify the phylogenetic position of Tosa-jidori based on its complete mitogenome, we constructed a ML tree (Figure 2, Supplementary Figure S2) using the mitogenome of Tosa-jidori along with 85 mitogenomes from NCBI and 47 D-loop sequences (five from this study and 42 from Oka et al [6]). We found that two individuals (Tosa 3 and Tosa 5: D-loop only) from our samples together with Oka et al [6] Tosa-jidori belong to Haplogroup D and the other individuals (Tosa 1, Tosa 4, and Tosa 6: D-loop only) along with Tosa 2 (complete mitogenome) from our samples were grouped with Sub-haplogroup E1 (Figure 2, Supplementary Figure S2). The MP tree showed essentially identical branching patterns to those of the ML tree (data not shown).

The topologies of phylogenetic trees based on the mitoge nome data (Figure 2) and the D-loop data (Figure 1) are fundamentally in agreement regarding the position of Tosa-Jidori. On both trees, we found that two individuals (Tosa 3 and Tosa 5) were located in Haplogroup D with the Tosa-jidori of Oka et al [6], and four individuals (Tosa 1, Tosa 2, Tosa 4, and Tosa 6) grouped with Sub-haplogroup E1.

Sub-haplogroup (E1) is globally distributed in domestic chickens, and commonly observed in South Asia to Europe as well as in commercial chicken lines. On the other hand, Haplogroup D is mainly distributed in Southeast Asia [13], and Oka et al [6] therefore suggested the Southeast Asian origin for Tosa-jidori. However, since the sub-haplogroup E1 is rare in Southeast Asia [13,18], our finding does not strongly support Southeast Asia being the sole origin of this breed.

Haplogroup E is widely considered to have originated from the Indian subcontinent and is now prevalent in chicken populations worldwide [7,10–13,22,25–28]. There are non-negligible proportions of Sub-haplogroup E1 found in East China, North China and Northeast China (geographically close to Japan), as reported in a previous study [13]. Therefore, taking account of the historical trading of Japan in ancient times [2], East Asia is the possible candidate of the origin of Tosa-jidori. However, since Miao et al [13] also reported that there is about 5% to 10% of Haplogroup E in the Southeast Asian continental region and about 20% in the Pacific islands, the Southeast Asian origin hypothesis for Tosa-jidori cannot be excluded. Interestingly, Haplogroup D, which is abundant in the Pacific, Southeast Asia and East Africa, is thought to be associated with the propagation of the culture of cockfighting, but rare in other regions and especially so in East Asia except for Japan [10–13,29]. Investigating how and when this Haplogroup D was introduced into Japan will shed light on the enigmatic history of JNCs.

In addition, only one mitogenome of any JNC breed (Silkie or Ukokkei) has been reported prior to this study [22]. The Silkie breed habituated in Japan before the beginning of the Edo period (1603 through 1867) [30]; it has many genetic features distinct from those observed in other chicken breeds, and does not belong to any of the three major JNC groups [2]. Therefore, this is the first study of the mitogenome from one of the three major JNC groups.

In conclusion, this study indicates the complicated origin and breeding history of Tosa-Jidori and indeed the JNC breeds. For in-depth unraveling of the origin and evolution of JNCs, more analyses of genome-wide markers as well as more samples covering all breeds are required.
