# Supplementary material for: Complete mitochondrial genome sequence of Tosa-Jidori sheds light on the origin and evolution of Japanese native chickens
Source: Anim Biosci. 2020 Apr 13;34(6):941–8. doi: 10.5713/ajas.19.0932 (PMC8100483; doi:10.5713/ajas.19.0932)
Supplement: Supplementary file 1 [file ajas-19-0932-suppl.pdf]

Table S1. List of sequences obtained from GenBank used in the phylogenetic analysis

	Source	Species/subspecies	Accession no.	Haplotype**	Haplogroup***	Location	Reference
1.	domestic chicken	*G. g. domesticus*	GU261684		A	China: Yunnan	Miao *et al*. (2013)
2.	wild fowl	*G. g. spadiceus*	GU261695		A	China: Yunnan	Miao *et al*. (2013)
3.	domestic chicken	*G. g. domesticus*	GU261700		A	Myanmar	Miao *et al*. (2013)
4.	domestic chicken	*G. g. domesticus*	AB086102		A	Japan: Hiroshima	Wada *et al*. (2004)
5.	domestic chicken	*G. g. domesticus*	GU261699		B	China: Yunnan	Miao *et al*. (2013)
6.	wild fowl	*G. g. spadiceus*	GU261704		B	China: Yunnan	Miao *et al*. (2013)
7.	domestic chicken	*G. g. domesticus*	GU261705		B	China: Yunnan	Miao *et al*. (2013)
8.	domestic chicken	*G. g. domesticus*	GU261714		B	China: Yunnan	Miao *et al*. (2013)
9.	wild fowl	*G. g. spadiceus*	AP003321		B	Laos: Vientiane	Nishibori *et al*. (2005)
10.	domestic chicken	*G. g. domesticus*	GU261675		C1	China: Hunan	Miao *et al*. (2013)
11.	domestic chicken	*G. g. domesticus*	GU261679		C1	China: Henan	Miao *et al*. (2013)
12.	domestic chicken	*G. g. domesticus*	GU261681		C1	China: Hunan	Miao *et al*. (2013)
13.	domestic chicken	*G. g. domesticus*	GU261701		C1	China: Henan	Miao *et al*. (2013)
14.	domestic chicken	*G. g. domesticus*	GU261718		C1	China: Yunnan	Miao *et al*. (2013)
15.	domestic chicken	*G. g. domesticus*	GU261680		C2	Southern India	Miao *et al*. (2013)
16.	wild fowl	*G. g. murghi*	GU261707		C3	India	Miao *et al*. (2013)
17.	wild fowl	*G. g. spadiceus*	GU261716		C3	Myanmar	Miao *et al*. (2013)
18.	domestic chicken	*G. g. domesticus*	GU261682		D1	Laos	Miao *et al*. (2013)
19.	domestic chicken	*G. g. domesticus*	GU261687		D1	Laos	Miao *et al*. (2013)
20.	wild fowl	*G. g. bankiva*	AP003323		D1	Indonesia: Bali	Nishibori *et al*. (2005)
21.	wild fowl	*G. g. gallus*	AP003322		D1	Philippine: Manila	Nishibori *et al*. (2005)
22.	domestic chicken	*G. g. domesticus*	GU261683		D2	China: Xinjiang	Miao *et al*. (2013)
23.	domestic chicken	*G. g. domesticus*	GU261677		D3	China: Zhejiang	Miao *et al*. (2013)
24.	domestic chicken	*G. g. domesticus*	GU261685		D3	Northeast India	Miao *et al*. (2013)
25.	domestic chicken	*G. g. domesticus*	GU261697		D3	Southern India	Miao *et al*. (2013)
26.	domestic chicken	*G. g. domesticus*	GU261686		E1	China: Henan	Miao *et al*. (2013)
27.	domestic chicken	*G. g. domesticus*	GU261694		E1	China: Hebei	Miao *et al*. (2013)
28.	wild fowl	*G. g. murghi*	GU261709		E1	India	Miao *et al*. (2013)
29.	domestic chicken	*G. g. domesticus*	GU261712		E1	China: Yunnan	Miao *et al*. (2013)
30.	domestic chicken	*G. g. domesticus*	GU261713		E1	China: Yunnan	Miao *et al*. (2013)
31.	domestic chicken	*G. g. domesticus*	HQ857210		E1	Northeast India	Miao *et al*. (2013)
32.	domestic chicken	*G. g. domesticus*	AP003319		E1	Laos: Vientiane	Nishibori *et al*. (2005)
33.	domestic chicken	*G. g. domesticus*	AP003317		E1	Commercial Line/WLCB	Nishibori *et al*. (2003)
34.	domestic chicken	*G. g. domesticus*	AY235571		E1	Commercial Lines	Froman and Kirby (2005)

Table S1.(continued)

35.	domestic chicken	*G. g. domesticus*	AP003318		E1	Commercial Line/WR	Nishibori *et al*. (2003)
36.	domestic chicken	*G. g. domesticus*	AY235570		E1	Commercial Line	Froman and Kirby (2005)
37.	domestic chicken	*G. g. domesticus*	AP003580		E1	Commercial Line/Cornell-P inbred line	Nishibori *et al*. (2003)
38.	domestic chicken	*G. g. domesticus*	HQ857209		E2	Northeast India	Miao *et al*. (2013)
39.	wild fowl	*G. g. murghi*	GU261708		E3	India	Miao *et al*. (2013)
40.	domestic chicken	*G. g. domesticus*	HQ857212		E3	Northeast India	Miao *et al*. (2013)
41.	domestic chicken	*G. g. domesticus*	HQ857211		E3	Northeast India	Miao *et al*. (2013)
42.	domestic chicken	*G. g. domesticus*	GU261688		F	China: Yunnan	Miao *et al*. (2013)
43.	domestic chicken	*G. g. domesticus*	GU261689		F	China: Yunnan	Miao *et al*. (2013)
44.	domestic chicken	*G. g. domesticus*	GU261691		F	Myanmar	Miao *et al*. (2013)
45.	wild fowl	*G. g. spadiceus*	GU261702		F	China: Yunnan	Miao *et al*. (2013)
46.	wild fowl	*G. g. spadiceus*	GU261703		F	Myanmar	Miao *et al*. (2013)
47.	domestic chicken	*G. g. domesticus*	GU261711		F	China: Yunnan	Miao *et al*. (2013)
48.	domestic chicken	*G. g. domesticus*	GU261717		F	China: Yunnan	Miao *et al*. (2013)
49.	domestic chicken	*G. g. domesticus*	DQ648776		F	China: Yunnan	Tong *et al*. (2006)
50.	domestic chicken	*G. g. domesticus*	GU261676		G	China: Yunnan	Miao *et al*. (2013)
51.	domestic chicken	*G. g. domesticus*	GU261678		G	China: Henan	Miao *et al*. (2013)
52.	wild fowl	*G. g. spadiceus*	GU261690		G	China: Yunnan	Miao *et al*. (2013)
53.	domestic chicken	*G. g. domesticus*	GU261710		G	China: Yunnan	Miao *et al*. (2013)
54.	domestic chicken	*G. g. domesticus*	GU261719		G	China: Yunnan	Miao *et al*. (2013)
55.	domestic chicken	*G. g. domesticus*	GU261715		H	China: Yunnan	Miao *et al*. (2013)
56.	domestic chicken	*G. g. domesticus*	GU261698		I	Northeast India	Miao *et al*. (2013)
57.	wild fowl	*G. g. spadiceus*	GU261706		W	China: Yunnan	Miao *et al*. (2013)
58.	wild fowl	*G. g. spadiceus*	GU261692		X	China: Yunnan	Miao *et al*. (2013)
59.	wild fowl	*G. g. spadiceus*	GU261693		Y	China: Yunnan	Miao *et al*. (2013)
60.	wild fowl	*G. g. jabouillei*	GU261674		Z	China: Hainan	Miao *et al*. (2013)
61.	wild fowl	*G. g. jabouillei*	GU261696		Z	China: Hainan	Miao *et al*. (2013)
62.	domestic chicken	*G. g. domesticus*	AB268506	A01	E1	Japan	Oka *et al* (2007)
63.	domestic chicken	*G. g. domesticus*	AB268507	A02	E1	Japan	Oka *et al* (2007)
64.	domestic chicken	*G. g. domesticus*	AB268508	A03	E1	Japan	Oka *et al* (2007)
65.	domestic chicken	*G. g. domesticus*	AB268509	A04	E1	Japan	Oka *et al* (2007)
66.	domestic chicken	*G. g. domesticus*	AB268510	A05	E1	Japan	Oka *et al* (2007)
67.	domestic chicken	*G. g. domesticus*	AB268511	A06	E1	Japan	Oka *et al* (2007)
68.	domestic chicken	*G. g. domesticus*	AB268512	A07	E1	Japan	Oka *et al* (2007)

Table S1.(continued)

69.	domestic chicken	*G. g. domesticus*	AB268513	A08	E1	Japan	Oka *et al* (2007)
70.	domestic chicken	*G. g. domesticus*	AB268514	A09	E1	Japan	Oka *et al* (2007)
71.	domestic chicken	*G. g. domesticus*	AB268515	A10	E1	Japan	Oka *et al* (2007)
72.	domestic chicken	*G. g. domesticus*	AB268516	B01	A	Japan	Oka *et al* (2007)
73.	domestic chicken	*G. g. domesticus*	AB268517	B02	A	Japan	Oka *et al* (2007)
74.	domestic chicken	*G. g. domesticus*	AB268518	B03	A	Japan	Oka *et al* (2007)
75.	domestic chicken	*G. g. domesticus*	AB268519	B04	A	Japan	Oka *et al* (2007)
76.	domestic chicken	*G. g. domesticus*	AB268520	B05	A	Japan	Oka *et al* (2007)
77.	domestic chicken	*G. g. domesticus*	AB268521	B06	A	Japan	Oka *et al* (2007)
78.	domestic chicken	*G. g. domesticus*	AB268522	C01	D	Japan	Oka *et al* (2007)
79.	domestic chicken	*G. g. domesticus*	AB268523	C02	D	Japan	Oka *et al* (2007)
80.	domestic chicken	*G. g. domesticus*	AB268524	C03	D	Japan	Oka *et al* (2007)
81.	domestic chicken	*G. g. domesticus*	AB268525	C04	D	Japan	Oka *et al* (2007)
82.	domestic chicken	*G. g. domesticus*	AB268526	C05	D	Japan	Oka *et al* (2007)
83.	domestic chicken	*G. g. domesticus*	AB268527	C06	D	Japan	Oka *et al* (2007)
84.	domestic chicken	*G. g. domesticus*	AB268528	C07	D	Japan	Oka *et al* (2007)
85.	domestic chicken	*G. g. domesticus*	AB268529	C08	D	Japan	Oka *et al* (2007)
86.	domestic chicken	*G. g. domesticus*	AB268530	D01	C1	Japan	Oka *et al* (2007)
87.	domestic chicken	*G. g. domesticus*	AB268531	D02	C1	Japan	Oka *et al* (2007)
88.	domestic chicken	*G. g. domesticus*	AB268532	D03	C1	Japan	Oka *et al* (2007)
89.	domestic chicken	*G. g. domesticus*	AB268533	D04	C1	Japan	Oka *et al* (2007)
90.	domestic chicken	*G. g. domesticus*	AB268534	D05	C1	Japan	Oka *et al* (2007)
91.	domestic chicken	*G. g. domesticus*	AB268535	D06	C1	Japan	Oka *et al* (2007)
92.	domestic chicken	*G. g. domesticus*	AB268536	D07	C1	Japan	Oka *et al* (2007)
93.	domestic chicken	*G. g. domesticus*	AB268537	D08	C1	Japan	Oka *et al* (2007)
94.	domestic chicken	*G. g. domesticus*	AB268538	D09	C1	Japan	Oka *et al* (2007)
95.	domestic chicken	*G. g. domesticus*	AB268539	E01	B	Japan	Oka *et al* (2007)
96.	domestic chicken	*G. g. domesticus*	AB268540	E02	B	Japan	Oka *et al* (2007)
97.	domestic chicken	*G. g. domesticus*	AB268541	E03	B	Japan	Oka *et al* (2007)
98.	domestic chicken	*G. g. domesticus*	AB268542	E04	B	Japan	Oka *et al* (2007)
99.	domestic chicken	*G. g. domesticus*	AB268543	F01	H	Japan	Oka *et al* (2007)
100.	domestic chicken	*G. g. domesticus*	AB268544	F02	H	Japan	Oka *et al* (2007)
101.	domestic chicken	*G. g. domesticus*	AB268545	G01	C*	Japan	Oka *et al* (2007)
102.	domestic chicken	*G. g. domesticus*	AB294232	B07	A	Japan	Oka *et al* (2007)
103.	domestic chicken	*G. g. domesticus*	AB294233	B08	A	Japan	Oka *et al* (2007)

Table S1.(continued)

104.	domestic chicken	*G. g. domesticus*	KJ778617	A	China	Direct Submission
105.	domestic chicken	*G. g. domesticus*	KM886936	A	Hunan: China	Direct Submission
106.	domestic chicken	*G. g. domesticus*	NC_001323	A	USA	Desjardins and Morais (1990)
107.	domestic chicken	*G. g. domesticus*	KF981434	A	Hunan: China	Liu *et al*. (2016)
108.	domestic chicken	*G. g. domesticus*	KM886937	B	Hunan: China	Direct submission
109.	domestic chicken	*G. g. domesticus*	KP742951	B	Beijing: China	Direct submission
110.	domestic chicken	*G. g. domesticus*	KP681581	B	Guangxi: China	Direct submission
111.	domestic chicken	*G. g. domesticus*	KM433666	B	Guangxi: China	Direct submission
112.	domestic chicken	*G. g. domesticus*	KM096864	B	Guangdong: China	Direct submission
113.	domestic chicken	*G. g. domesticus*	KP269069	C1	Guangxi: China	Direct submission
114.	domestic chicken	*G. g. domesticus*	KP681580	C1	Guangxi: China	Direct submission
115.	domestic chicken	*G. g. domesticus*	KF939304	D1	Hunan: China	Direct submission
116.	domestic chicken	*G. g. domesticus*	KP211424	G	India	Direct submission
117.	domestic chicken	*G. g. domesticus*	KP211422	I	India	Direct submission
118.	domestic chicken	*G. g. domesticus*	KP211418	E3	India	Direct submission
119.	domestic chicken	*G. g. domesticus*	KP211419	E3	India	Direct submission
120.	domestic chicken	*G. g. domesticus*	KP211420	E2	India	Direct submission
121.	domestic chicken	*G. g. domesticus*	KF826490	E1	Hunan: China	Direct submission
122.	domestic chicken	*G. g. domesticus*	KF954727	E1	Hunan: China	Direct submission
123.	domestic chicken	*G. g. domesticus*	KP211421	E1	India	Direct submission
124.	wild fowl	*G. g. gallus*	KP211423	E1	India	Direct submission
125.	domestic chicken	*G. g. domesticus*	KP211425	E1	India	Direct submission
126.	domestic chicken	*G. g. domesticus*	KP244335	E1	Hunan: China	Direct submission
127.	domestic chicken	*G. g. domesticus*	KR347464	E1	Beijing: China	Direct submission

C*: Unknown sub-haplotype within haplogroup C based on the the maximum likelihood tree inferred in this study based on the mitogenome data (Figure S2).

Haplotype**: Haplotypes defined by Oka *et al.* (2007). The haplotype names were indicated only for Oka et al. (2007)'s sequence data.

Haplogroup***: Haplogroups were followed to the definition by Miao *et al.* (2013). As for sequence 62~127, the haplogroups were determined by the maximum likelihood tree inferred in this study based on the mitogenome data (Figure S2).

References:

Desjardins P, Morais R (1990) Sequence and gene organization of the chicken mitochondrial genome. A novel gene order in higher vertebrates. J Mol Biol 212(4):599-634.

Froman DP, Kirby JD (2005) Sperm mobility: phenotype in roosters (*Gallus domesticus*) determined by mitochondrial function. Biol Reprod 72(3):562-567.

Liu LL, Xie HB, Yu QF, He SP, He JH (2016) Determination and analysis of the complete mitochondrial genome sequence of Taoyuan chicken. Mitochondrial DNA Part A, 27(1): 371-372

Miao YW, Peng MS, Wu GS, Ouyang YN, Yang ZY, Yu N, Liang JP, Pianchou G, Beja-Pereira A, Mitra B, Palanichamy MG, Baig M, Chaudhuri TK, Shen YY, Kong QP, Murphy RW, Yao YG, Zhang YP (2013) Chicken domestication: an updated perspective based on mitochondrial genomes. Heredity, 110: 277–282. 2013.

Nishibori M, Shimogiri T, Hayashi T, Yasue H (2005) Molecular evidence for hybridization of species in the genus *Gallus* except for *Gallus varius*. Anim Genet 36(5):367-375.

Nishibori M, Hanazono M, Yamamoto Y, Tsudzuki M, Yasue H (2003) Complete nucleotide sequence of mitochondrial DNA in White Leghorn and White Plymouth Rock chickens. Anim Sci J 74(5):437-439.

Oka T, Ino Y, Nomura K, Kawashima S. Kuwayama T. Hanada H. Amano T. Takada M. Takahata N. Hayashi Y. Akishinonomiya F. Analysis of mtDNA sequences shows Japanese native chickens have multiple origins. Anim. Genet. 2007; 38: 287-293. https://doi. 10.1111/j.1365-2052.2007.01604.x

Tong XM, Liang Y, Wang W, Xu SQ, Zheng XG, Wang J, Yu J. (2006) Complete sequence and gene organization of the Tibetan chicken mitochondrial genome. Hereditas (Beijing) 28(7):769-777.

Wada Y, Yamada Y, Nishibori M, Yasue H (2004) Complete nucleotide sequence of mitochondrial genome in silkie fowl (*Gallus gallus* var. *domesticus*). J Poult Sci 41(1):76-82.

Table S2. Sequence variation of 6 individuals of Tosa-Jidori breed observed in the mt DNA D-loop region

							Strain name	GenBank accession number
						1		
	2	2	3	3	4	2		
	1	8	0	3	4	1		
	7	1	6	0	6	3		
Ref. sequence	C	A	T	C	T	T		
Tosa 1	·	·	·	.	·	·	910	LC507812
Tosa 2	·	·	·	T	·	·	921	LC507813
Tosa 3	T	G	C	.	C	C	911	LC507814
Tosa 4	·	·	·	.	·	·	922	LC507815
Tosa 5	T	G	C	.	C	C	51	LC507816
Tosa 6	·	·	·	T	·	·	Tsuchimoto	LC507817

Vertically oriented numbers indicate the nucleotide position. Mutations are scored relative to the reference sequence (white leghorn CB line, GenBank accession No. AP003317; Nishibori *et al*., 2003). Dots (.) indicate identity with the reference sequence.

Reference

Nishibori M, Hanazono M, Yamamoto Y, Tsudzuki M, Yasue H (2003) Complete nucleotide sequence of mitochondrial DNA in White Leghorn and White Plymouth Rock chickens. Anim Sci J 74(5):437-439.

Figure S1: A ML phylogenetic tree of chickens (the log-likelihood score is -2846.2) based on complete mitochondrial D-loop sequences of 133 individuals. The gaps were treated as missing nucleotides, and all sites (1,233 bp) were included in this analysis. Nodal numbers indicate the bootstrap values with 1,000 replications. The bootstrap values >50% are indicated. The names of OTUs are indicated by NCBI accession numbers. Haplotype names defined by Oka et al. (2007) are also indicated in parentheses after the relevant accession numbers. Nomenclature of the haplogroups follows Miao et al. (2013). Six sequences of Tosa-Jidori (in this study) as well as Tosa-Jidori studied by Oka et al. (2007) (accession number AB268523-C02) are indicated by green and blue arrows, respectively. The branch lengths are proportional to the nucleotide substitution rate. However, the branches of haplogroups C* and I are partially omitted due to their long branch lengths.

Figure 2: A ML phylogenetic tree of chickens (the log-likelihood score is -27799.9). The gaps were dealt as the missing nucleotide, and all sites (16,797 bp) were involved in this analysis. Nodal numbers indicate the bootstrap values with 1,000 replications. The bootstrap values >50% are indicated. Complete mitogenome sequences are indicated by solid lines and the shorter complete mitochondrial D-loop sequences are indicated by thin dashed lines. The names of OTUs are indicated by NCBI accession numbers. Haplotype names defined by Oka et al. (2007) are also indicated in parentheses after the relevant accession numbers. Nomenclature of the haplogroups follows Miao et al. (2013). Six sequences of Tosa-Jidori (in this study) as well as Tosa-Jidori studied by Oka et al. (2007) (accession number AB268523-C02) are indicated by green and blue arrows, respectively. The branch lengths are proportional to the nucleotide substitution rate.

References

Oka T, Ino Y, Nomura K, Kawashima S.　Kuwayama T.　Hanada H.　Amano T.　Takada M.　Takahata N.　Hayashi Y.　Akishinonomiya F. Analysis of mtDNA sequences shows Japanese native chickens have multiple origins. Anim. Genet. 2007; 38: 287-293. https://doi. 10.1111/j.1365-2052.2007.01604.x

Miao YW, Peng MS, Wu GS, Ouyang YN, Yang ZY, Yu N, Liang JP, Pianchou G, Beja-Pereira A, Mitra B, Palanichamy MG, Baig M, Chaudhuri TK, Shen YY, Kong QP, Murphy RW, Yao YG, Zhang YP (2013) Chicken domestication: an updated perspective based on mitochondrial genomes. Heredity, 110: 277–282. 2013.

Figure S1

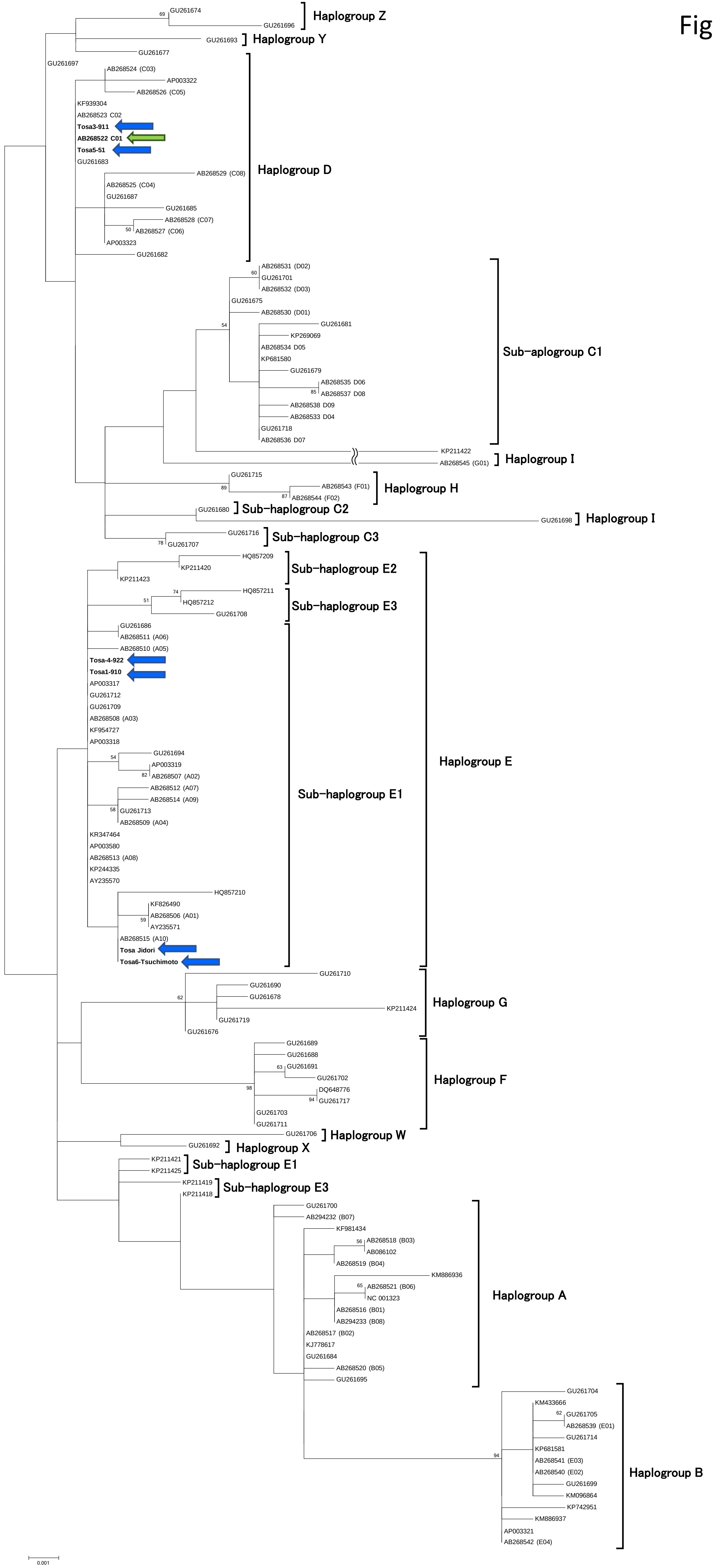

Haplogroup Z
Haplogroup Y
Haplogroup D
Sub-aplogroup C1
Haplogroup I
Haplogroup H
Sub-haplogroup C2
Sub-haplogroup C3
Sub-haplogroup E2
Sub-haplogroup E3
Sub-haplogroup E1
Haplogroup E
Haplogroup G
Haplogroup F
Haplogroup W
Haplogroup X
Haplogroup A
Haplogroup B
Tosa3-911
AB268522 C01
Tosa5-51
Tosa-4-922
Tosa1-910
Tosa Jidori
Tosa6-Tsuchimoto
0.001

Figure S2

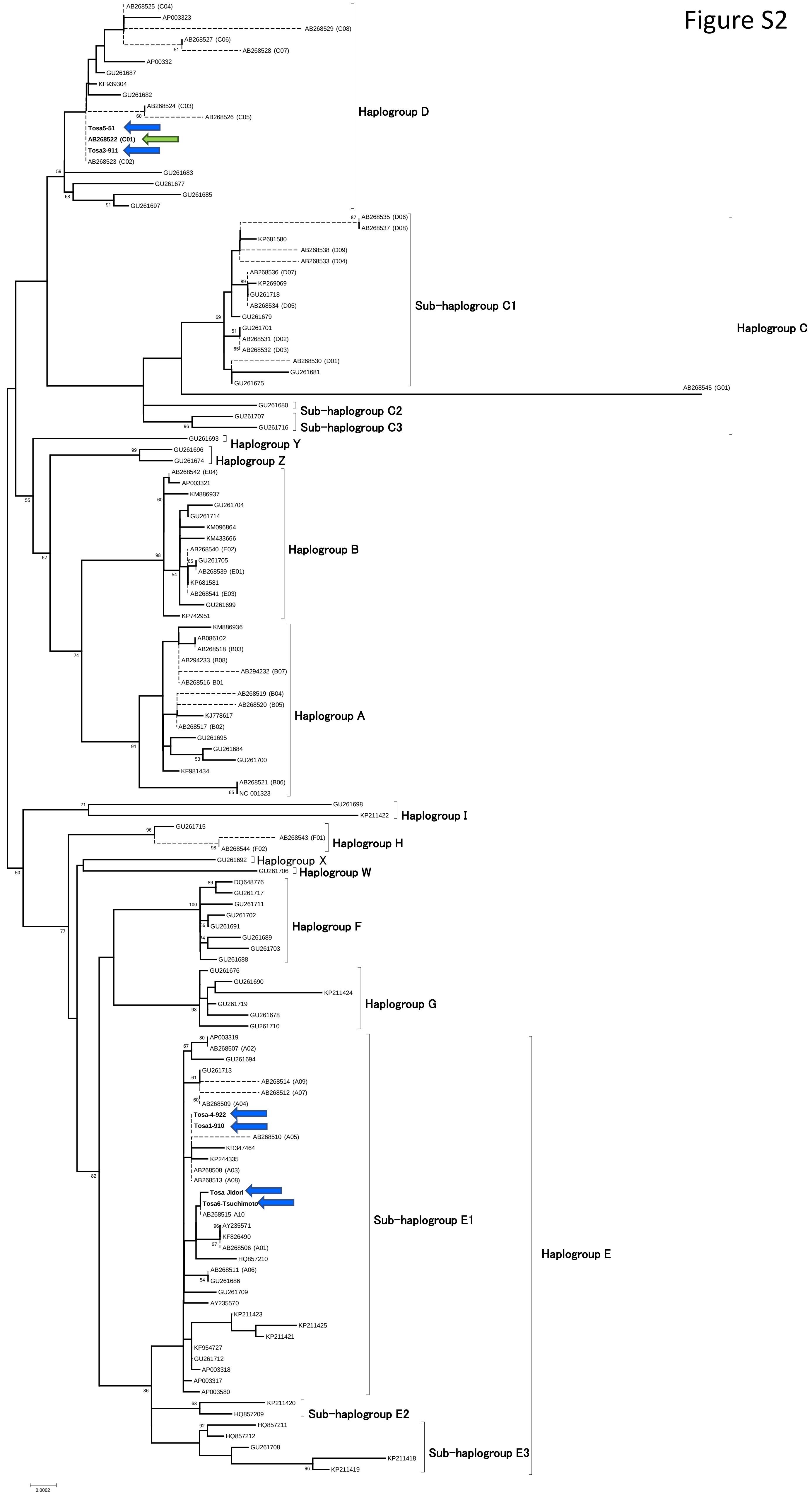

AB268525 (C04)
AP003323
AB268529 (C08)
AB268527 (C06)
AB268528 (C07)
AP00332
GU261687
KF939304
GU261682
AB268524 (C03)
AB268526 (C05)
Tosa5-51
AB268522 (C01)
Tosa3-911
AB268523 (C02)
GU261683
GU261677
GU261685
GU261697
Haplogroup D
AB268535 (D06)
AB268537 (D08)
KP681580
AB268538 (D09)
AB268533 (D04)
AB268536 (D07)
KP269069
GU261718
AB268534 (D05)
GU261679
GU261701
AB268531 (D02)
AB268532 (D03)
AB268530 (D01)
GU261681
GU261675
Sub-haplogroup C1
AB268545 (G01)
Haplogroup C
GU261680
Sub-haplogroup C2
GU261707
GU261716
Sub-haplogroup C3
GU261693
Haplogroup Y
GU261696
GU261674
Haplogroup Z
AB268542 (E04)
AP003321
KM886937
GU261704
GU261714
KM096864
KM433666
AB268540 (E02)
GU261705
AB268539 (E01)
KP681581
AB268541 (E03)
GU261699
KP742951
Haplogroup B
KM886936
AB086102
AB268518 (B03)
AB294233 (B08)
AB294232 (B07)
AB268516 B01
AB268519 (B04)
AB268520 (B05)
KJ778617
AB268517 (B02)
GU261695
GU261684
GU261700
KF981434
AB268521 (B06)
NC 001323
Haplogroup A
GU261698
KP211422
Haplogroup I
GU261715
AB268543 (F01)
AB268544 (F02)
Haplogroup H
GU261692
Haplogroup X
GU261706
Haplogroup W
DQ648776
GU261717
GU261711
GU261702
GU261691
GU261689
GU261703
GU261688
Haplogroup F
GU261676
GU261690
KP211424
GU261719
GU261678
GU261710
Haplogroup G
AP003319
AB268507 (A02)
GU261694
GU261713
AB268514 (A09)
AB268512 (A07)
AB268509 (A04)
Tosa-4-922
Tosa1-910
AB268510 (A05)
KR347464
KP244335
AB268508 (A03)
AB268513 (A08)
Tosa Jidori
Tosa6-Tsuchimoto
AB268515 A10
AY235571
KF826490
AB268506 (A01)
HQ857210
AB268511 (A06)
GU261686
GU261709
AY235570
KP211423
KP211425
KP211421
KF954727
GU261712
AP003318
AP003317
AP003580
Sub-haplogroup E1
KP211420
HQ857209
Sub-haplogroup E2
HQ857211
HQ857212
GU261708
KP211418
KP211419
Sub-haplogroup E3
Haplogroup E
0.0002
